# Effect of Elevated Atmospheric CO_2_ and Temperature on the Disease Severity of Rocket Plants Caused by Fusarium Wilt under Phytotron Conditions

**DOI:** 10.1371/journal.pone.0140769

**Published:** 2015-10-15

**Authors:** Walter Chitarra, Ilenia Siciliano, Ilario Ferrocino, Maria Lodovica Gullino, Angelo Garibaldi

**Affiliations:** 1 Centre for Innovation in the Agro-Environmental Sector, AGROINNOVA, University of Torino, Largo P. Braccini 2, Grugliasco (TO), Italy; 2 Department of Agricultural, Forest and Food Sciences (DISAFA), University of Torino, Largo P. Braccini 2, Grugliasco (TO), Italy; National University of Ireland - Galway, IRELAND

## Abstract

The severity of *F*. *oxysporum* f.sp. *conglutinans* on rocket plants grown under simulated climate change conditions has been studied. The rocket plants were cultivated on an infested substrate (4 log CFU g^-1^) and a non-infested substrate over three cycles. Pots were placed in six phytotrons in order to simulate different environmental conditions: 1) 400–450 ppm CO_2_, 18–22°C; 2) 800–850 ppm CO_2_, 18–22°C; 3) 400–450 ppm CO_2_, 22–26°C, 4) 800–850 ppm CO_2_, 22–26°C, 5) 400–450 ppm CO_2_, 26–30°C; 6) 800–850 ppm CO_2_, 26–30°C. Substrates from the infested and control samples were collected from each phytotron at 0, 60 and 120 days after transplanting. The disease index, microbial abundance, leaf physiological performances, root exudates and variability in the fungal profiles were monitored. The disease index was found to be significantly influenced by higher levels of temperature and CO_2_. Plate counts showed that fungal and bacterial development was not affected by the different CO_2_ and temperature levels, but a significant decreasing trend was observed from 0 up to 120 days. Conversely, the *F*. *oxysporum* f.sp. *conglutinans* plate counts did not show any significantly decrease from 0 up to 120 days. The fungal profiles, evaluated by means of polymerase chain reaction denaturing gradient gel electrophoresis (PCR-DGGE), showed a relationship to temperature and CO_2_ on fungal diversity profiles. Different exudation patterns were observed when the controls and infested plants were compared, and it was found that both CO_2_ and temperature can influence the release of compounds from the roots of rocket plants. In short, the results show that global climate changes could influence disease incidence, probably through plant-mediated effects, caused by soilborne pathogens.

## Introduction

Nowadays, how climate changes will influence plant-pathogen interactions and their impact on production is largely debated and represents a challenge for future programmes focused on disease management under global change conditions [1]. An increasing numbers of multidisciplinary approaches have reported the effects of rising temperature and CO_2_ levels on crop productions and physiological changes [2]. On the other hand, researchers have only recently focused on plant disease prediction and management under climate changes [3]. Plant pathogens represents an important constraint for the security of our future food as a consequence of population increases, urbanization, globalization and changes in climate [4]. Moreover, several pathogens produce toxins and other compounds that are dangerous to human and animal health which could affect market and world trade. On these grounds, a multidisciplinary approach is needed to implement predictive models and include higher levels of ecological interactions [5]. As recently reported by an Intergovernmental Panel on Climate Changes [6], anthropogenic emissions of greenhouse gases have caused negative impacts on human and natural systems over the last three decades. In other words, changes in the atmospheric composition and temperature as well as humidity alterations can have an impact on host plant physiology and influence a plant’s resistance against pathogens [7–9]. On one hand, rises in CO_2_ and temperature can affect the sense and responses of a plant by increasing the photosynthesis rates, water and light-use efficiency, leaf surface properties, changes in anatomy, morphology, phenology and root exudates, thus profoundly modifying native plants and soil microbial communities [1,3,8,10,11]. On the other hand, elevated CO_2_ influences the pathogenicity, host-pathogen interaction and epidemiology of fungal diseases [3,12–15,16]. An enlarged canopy coupled with a favourable microclimate offers more sites for infection and increases fungal pathogen fecundity, which has been shown to lead to twice the number of lesions for high CO_2_ concentrations [17,18]. Furthermore, the mutation and selection of plant pathogens and the consequent development of new strains have also been predicted [3,19].

Phytotron-based studies are optimized to study the interactions that occur between plants, soil and soil microbiota and they combine environmental parameters such as light intensity, CO_2_ concentrations, temperature, and relative humidity [20]. Phytotron chambers have in fact been largely used to study the effects of global changes on several pathosystems [11, 14–16,21]. Plants directly or indirectly affect the development of soil microbiota in the rhizosphere and in bulk soil by means of root exudates [22]. However, most studies conducted so far report contrasting results; such as a decrease [23], an increase [24] or no changes [25] in microbial diversity and activities. The development of soil microbial populations needs a multiphasic approach that can couple traditional microbial analysis with molecular tools such as polymerase chain reaction denaturing gradient gel electrophoresis (PCR-DGGE). This technique has been optimized to monitor soil microflora changes under climate change conditions [16,26,27]. *Fusarium oxysporum* and the related *formae specialis* cause greater economic damage to several crops than other plant pathogens [28]. Rocket (*Eruca sativa*) is a high-value horticultural crop, mainly cultivated in the Mediterranean area, up to 3–5 times in the same soil which is affected by emerging soilborne pathogens [20,29] and *Fusarium oxysporum* f.sp. *conglutinans* has recently been detected in Italy on cultivated (*Eruca sativa*) and wild rocket (*Diplotaxis tenuifolia*) [30].

The aim of this work was to study the effect of *F*. *oxysporum* f.sp. *conglutinans*, artificially infested in a growing substrate, on rocket plants grown under simulated climate change conditions with rising CO_2_ concentrations and temperatures in phytotron chambers. Six temperature/CO_2_ combinations were studied. Disease incidence, the physiological performances of leaves, microbial and fungal cultivable abundance and the main root exudate components were monitored to evaluate the effects of climatic changes on disease development over time. Furthermore, shifts in fungal communities were assessed using the DGGE technique on DNA directly extracted from an infested growing substrate.

## Materials and Methods

### Inoculum preparation

*F*. *oxysporum* f.sp. *conglutinans* (ATCC16600RB, Agroinnova, Grugliasco, Italy), which is resistant to benomyl [31], was used and cultured in 1000-mL Erlenmeyer flasks containing 250 mL of hydrolized casein. The flasks were incubated on a platform shaker at 20–25°C for 12 days. Chlamydospores were recovered by means of centrifugation for 20 min at 8000*g* at 20°C, following the removal of mycelia fragments by sieving through cheesecloth. The chlamydospore suspension was dried and mixed with sterile talc powder (1:2 w/w), as described by Locke & Colhoun [32], and stored at room temperature for further use. The number of chlamydospores per gram of talc was assessed by serial plating on a Komada medium [33] containing 10 mg L^-1^ of benomyl (Benlate, 50% a.i.; DuPont de Nemours, Milan, Italy). The talc formulation was incorporated into the soil to achieve the desired inoculum of 4 Log colony forming units (CFU) g^-1^.

### Plant material and experimental set-up

Two experimental trials were carried out at Agroinnova (Grugliasco, Itay). Plastic tanks containing 100L of a mixture (1:1 v/v) of peat-perlite substrate (Tecno2, Turco Silvestro sphagnum peat moss, Albenga, SV, Italy) and sandy loam soil (pH, 7.3; organic matter content, 2.2%; cation exchange capacity, 2.6 meq/100 g soil) were prepared. The final substrate was made up of: sand, 76%; silt, 14%; clay, 10%; pH, 7.51; organic matter content, 2,59% and had a cation exchange capacity of 5.99 meq/100 g soil and subjected to steam sterilization before use. The substrate was artificially inoculated with *F*. *oxysporum* f. sp. *conglutinans* (ATCC16600RB) to reach a final concentration of 4 Log CFU ml^-1^. A non-inoculated tank was used as the control.

*Eruca sativa* Mill seeds (cultivated rocket) were disinfected in a solution of 1% sodium hypocloride plus 0.01% of Tween-20, and then rinsed twice in water for 1 min. The seeds were air-dried at RT and stored at 4°C until use. The seeds were sown in a greenhouse in plug trays (20–26°C, 70% RH and natural light condition). After 15–20 days, the first seedling-leaves were developed. The rocket plants were left to grow under Phytotron conditions for 7 days. Subsequently, 48 pots (2L each) were prepared from the inoculated tank and another 48 pots were prepared from the non-inoculated tank and used as controls. The rocket plants were then transplanted (4 plants/pot), and 8 inoculated and 8 non-inoculated pots were placed in 6 different phytotrons (PGC 9.2, TECNO.EL, Italy). One replicate consisted of two pots. The rocket plants were kept in the phytotrons under six different temperature and CO_2_ combinations according to the following ranges: 1) 400–450 ppm CO_2_, 18–22°C; 2) 800–850 ppm CO_2_, 18–22°C; 3) 400–450 ppm CO_2_, 22–26°C, 4) 800–850 ppm CO_2_, 22–26°C, 5) 400–450 ppm CO_2_, 26–30°C; 6) 800–850 ppm CO_2_, 26–30°C. The temperatures, light and humidity were changed gradually during the day in order to simulate natural conditions. Three subsequent cultivation cycles were carried out in the same pot. Each crop cycle lasted 35–37 days after transplanting. The plants were irrigated daily in order to maintain the soil moisture at field capacity.

Substrate samples were taken at time 0 (immediately before plant transplanting) and after 60 and 120 days for microbial enumeration and molecular analyses (PCR-DGGE), while the physiological measurements of the plant leaves were conducted at the end of each rocket cycle.

### Disease incidence evaluation

The effectiveness of the different simulated climate change conditions on the severity of *F*. *oxysporum* f.sp. *conglutinans* on rocket was checked weekly by evaluating the pathogen development using a previously reported disease index [34]. Wilted plants were counted and removed and the final disease rating was made at the end of the experiment (35–37 days after transplanting). At the end of each cycle, re-isolation from infected plants on a Komada medium supplemented with 10 mg L^-1^ of benomyl was performed to confirm the presence of *F*. *oxysporum* as the causal agent of the observed symptoms. During the latter survey, the total fresh plant biomass was also rated using a technical balance (Orma SNC, Milano, Italy) to evaluate the effect of the treatments on plant growth.

### Physiological measurements of the leaves

In order to observe the effects of the climate change conditions on the leaf physiological activity of the infected and control rocket plants, the photosynthetic efficiency and chlorophyll content were monitored. Measurements were performed following the experimental protocol reported by Pugliese et al. [35], with only minor modifications.

The chlorophyll content index (CCI) was measured with the SPAD 502 chlorophyll meter (CCM-200, Opti-Sciences, Inc., Hudson, NH, USA), which determined the relative amount of chlorophyll in the leaf by measuring the absorbance in the red and near-infrared regions (650 and 940 nm, respectively). Chlorophyll meter readings were taken from each rocket plant in the second or third leaves (fully developed) from the top on ten randomly selected plants (one leaf/plant) at the end of each cultivation cycle.

The photosynthetic efficiency measurements were performed on five randomly selected leaves using a portable continuous-excitation type fluorimeter (Handy-PEA, Hansatech Instruments Ltd, Norfolk, UK), according to the manufacturer’s instructions, at the end of each cultivation cycle of rocket plants grown in infested and control substrates.

### Root exudate analyses

In order to investigate the relationship between climate changes and the turnover of low molecular weight organic compounds in the rhizosphere, water-soluble root exudates were collected and analyzed. A sterilized CaSO_4_ 0.01M solution (collection media) was used to collect the root exudates [36]. For each condition tested, three randomly selected rocket plants were taken from infested and not infested pots and the soil was carefully removed using deionized water, without damaging the roots. The plants were then placed in a 50 mL centrifuge tube with 15 mL of collection media for 2 hours in each phytotron. The collection media was then filtered through a 0.45 μm membrane filter. pH was measured and the samples were freeze dried and dissolved in 3 mL of ddH_2_O. Three main groups of organic substances were analyzed: total organic carbon (TOC), total organic acids and amino acid compounds.

TOC was determined by means of the colorimetric method. An aliquot of 1 mL was mixed with 2 mL of K_2_Cr_2_O_7_ (2 N) and 1 mL of H_2_SO_4_ concentrate and kept at 150°C for 10 min. The samples were cooled to room temperature and then analyzed by using a Spectroquant Pharo 300 spectrophotometer (Merck, Darmstadt, Germany) at λ = 585 nm. Glucose was used as the reference C substance. For the amino acids analysis, 1 mL of sample was mixed with 1 mL of borate buffer (0.4 M pH = 9.5) and 1 mL of dansyl chloride solution (Sigma-Aldrich, St. Louis, MO, USA) at 10 mg/mL. The samples were incubated at 65°C for 30 min [37]. The analysis was performed using an Agilent 1100 HPLC system (Agilent Technologies, Palo Alto, CA). Zorbax Eclipse Plus C18 (4.6x100 mm, 3.5 μm, Agilent Technologies) was used for the chromatographic separation of the amino acids with a linear gradient from 10 to 100% of acetonitrile (Merck) in 15 min. The derivatized amino acids were detected by means of a fluorescent detector with excitation at 335 nm and emission at 524 nm. A mixture of 20 standard amino acids (Sigma-Aldrich) was used as the reference. The organic acids were separated with a Synergi Hydro-RP column (4.6x250 mm, 4 μm, Phenomenex, Torrance, CA, USA) in an isocratic condition, using buffer phosphate (50 mM) as the mobile phase. The detector was set at λ = 214 nm. A mixture of standard organic acids (Sigma-Aldrich) was used as the reference.

### Microbial count

Substrate samples (25 grams) were collected from 8 pots for both infested and control treatments, at a depth of 2–5 cm, and were placed in sterilized polyethylene bags using a sterilized spatula. The samples were passed through a sieve to remove any vegetation, and mixed at room temperature for 30 min with 225 mL of quarter strength Ringer’s solution (Merck) in sterilized flasks on a rotary shaker (100 rpm). Decimal dilutions were prepared in quarter strength Ringer’s solution and aliquots of 1 ml of the appropriate dilutions were poured, in triplicate, onto the following media: plate count agar (PCA, Oxoid, Milan) incubated at 28°C for 48h for the total bacterial counts; potato dextrose agar (PDA, Merck) supplemented with streptomycin (0,5 g/L) and a Komada medium supplemented with 10 mg L^-1^ of benomyl (Benlate, 50% w.g., DuPont, USA), kept at 25°C for 7 days for the total fungal and *F*. *oxysporum* f.sp. *conglutinans* enumerations, respectively. The results were calculated as the mean of the Log counts of three independent determinations.

### Substrate DNA extraction and DGGE analysis

Infested substrate samples (250 mg) were taken from 4 pots/phytotron at 0, 60 and 120 days after transplanting for DNA extraction. DNA exctract according to the protocol described by the NucleoSpin® Soil manufacturer (Macherey-Nagel, Germany) and was quantified using the NanoDrop 1000 spectrophotometer (Thermo Scientific) and standardized at 10 ng μl^-1^. Fungal DNA was amplified using fungal ITS primers according to Gao *et al*. [38]. PCR products for the ITS region were analyzed by denaturing gradient gel electrophoresis (DGGE) at 25–35% using a Bio-Rad Dcode, as previously described [16].

### Statistical analysis

The data obtained from the plate counts, root exudates and leaf physiological measurements were analyzed using one-way analysis of variance (ANOVA), with treatments being the main factor. ANOVA analyses were performed with the SPSS 22.0 statistical software package (SPSS Inc., Cary, NC, USA). The Duncan HSD test was applied when ANOVA revealed significant differences (P < 0.05). A database of fingerprints was created using the PyElph software. A combined data matrix that included all the fingerprints for the ITS region was obtained, and dendrograms of similarity were retrieved using the Dice coefficient and the Unweighted Pair Group Method with the Arithmetic Average (UPGMA) clustering algorithm [39] utilizing PyElph software [40]. The similarity distance matrix generated through PyElph was used to build a Projection on Latent Structures—Discriminant Analysis (PLS-DA) utilizing “mixOmics” in the R environment (www.r-project.org). The obtained binary band-matching tables were considered to calculate the Shannon-Wiener diversity index (*H’*) [41] using PAST (PAleontological STatistics) software [42].

## Results

### Disease index assessment and fresh biomass production

The disease index (DI, 0–100) was found to be significantly influenced by the temperature and CO_2_ levels (Table 1). No symptoms were observed for the control plants in any of the cycles or phytotron conditions that were considered. Generally, higher levels of CO_2_ and temperature were factors that significantly influenced the DI in the infected plants in the simulated conditions. At 18–22°C, the DI was significantly lower for both CO_2_ ranges (P < 0.05) than the other conditions. Conversely, the DI significantly increased at 22–26°C, particularly for higher CO_2_ levels (P < 0.05) where a DI of 54 was reached. At 26–30°C, a slightly decreasing trend was recorded for both CO_2_ levels, although a higher DI (43,4) was observed at 800–850 ppm CO_2,_ with a significantly different value than that of the 18–22°C conditions (P < 0.05). The fresh weight (FW) data pertaining to the infected plants showed significant differences (P < 0.05) as a consequence of temperature, CO_2_ and disease development (Table 1). Lower FW values were obtained at 18–22°C for both CO_2_ conditions and in phytotrons 4 and 6 (800–850 ppm CO_2_, 22–26°C and 800–850 ppm CO_2_, 26–30°C, respectively) where disease development was higher (Table 1). The control plants showed a higher FW than the diseased rocket plants, with the exception of 18–22°C (phytotrons 1 and 2: 400–450 ppm CO_2_, 18–22°C and 800–850 ppm CO_2_, 18–22°C) in which FW was significantly lower (P < 0.05) than the other tested.

**Table 1:** Disease index (0–100) and total fresh weight of the plants at the end of the replicates (FW).

	Parameters	Phytotron ^a^					
		1	2	3	4	5	6
		(400-450ppm CO_2_ 18–22°C)	(800-850ppm CO_2_ 18–22°C)	(400-450ppm CO_2_ 22–26°C)	(800-850ppm CO_2_ 22–26°C)	(400-450ppm CO_2_ 26–30°C)	(800-850ppm CO_2_ 26–30°C)
Infected plants	Disease Index (0–100)	17.2 ± 9.4 a	18.80 ± 11.40 a	25.00 ± 12.5 ab	54.00 ± 12.20 c	22.90 ± 3.60 ab	43.40 ± 11.90 bc
	Fresh Weight (g plants^-1^)	3.43 ± 0.21 a	2.73 ± 0.29 a	12.33 ± 0.21 c	3.87 ± 0.06 a	14.45 ± 0.13 d	9.70 ± 0.18 b
Control plants	Disease Index (0–100)	0.00 a	0.00 a	0.00 a	0.00 a	0.00 a	0.00 a
	Fresh Weight (g plants^-1^)	3.80 ± 0.44 a	3.88 ± 0.21 a	13.33 ± 0.41 b	14.50 ± 0.63 b	16.27 ± 0.86 c	13.03 ± 0.46 b

^a^The disease index (0–100) and total plant fresh weight were expressed on the basis of three replicates. Values with different superscripts in the same row differ significantly according to Tukey's HSD test (P<0.05)

### Effect on the physiological performances of the leaves

**Fig 1:**
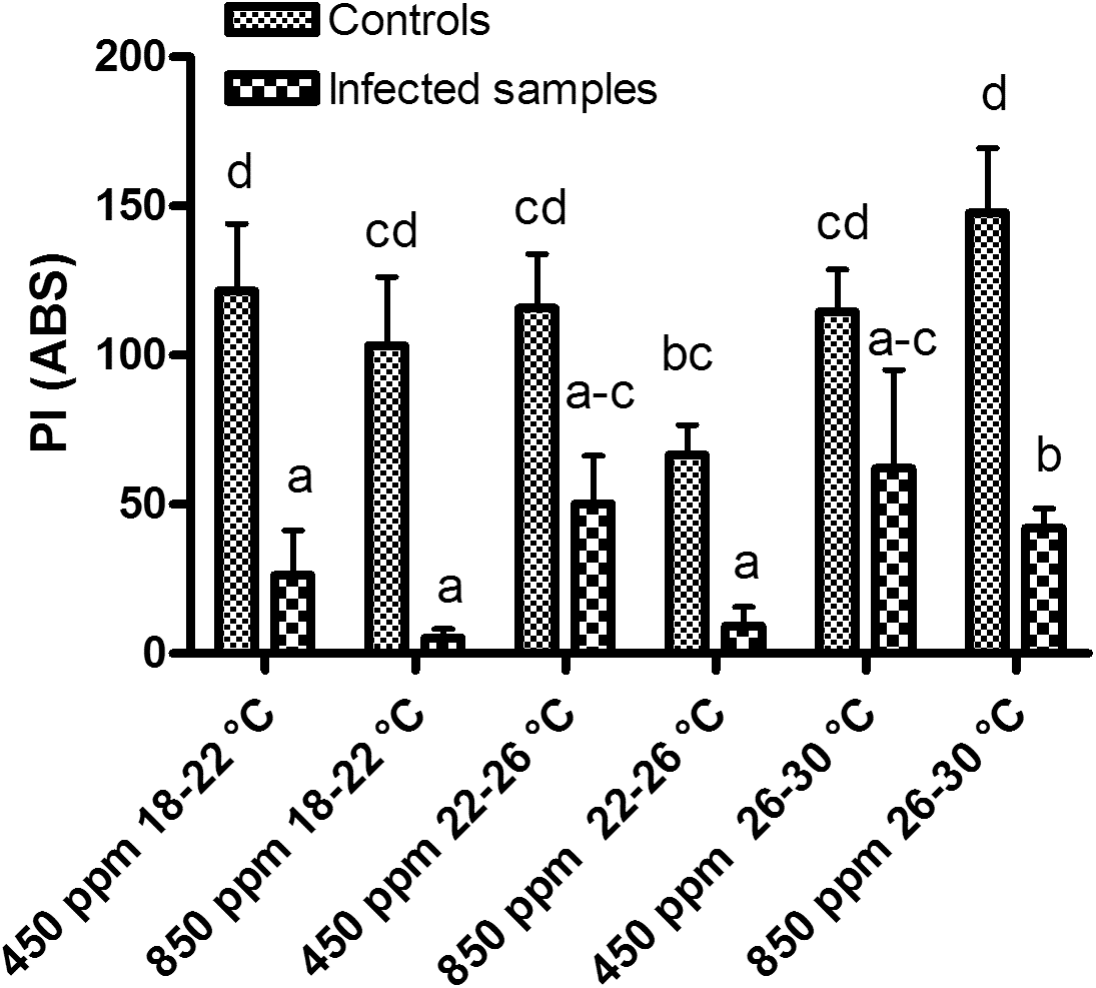
Photosynthetic efficiency measurements. Effect of different CO_2_ and temperature combinations on the photosynthetic efficiency of the leaves (PI) of rocket plants grown in a substrate artificially infested with *F*. *oxysporum* f.sp. *conglutinans* and the control. Tukey's HSD test (P < 0.05).

The photosynthetic efficiency index (PI) for the infected plants showed a particularly pronounced and statistically significant (P < 0.05) decreasing trend for all the conditions tested at the end of the cycles (Fig 1). In other words, PI appeared to be particularly affected by disease development (Table 1) coupled with the environmental conditions. Lower PI values (P < 0.05) were recorded for the infected plants from phytotrons 1, 2 and 4 (400–450 ppm CO_2_, 18–22°C; 800–850 ppm CO_2_, 18–22°C and 800–850 ppm CO_2_, 22–26°C, respectively). Moreover, the highest temperature provided the best evidence of impaired PI at high CO_2_ in the infected plants.

In general, the non-inoculated plants showed higher photosynthetic efficiency than the infected ones, with the exception of those from phytotron 4 (800–850 ppm CO_2_, 22–26°C) where PI was lower than for the other conditions.

**Fig 2:**
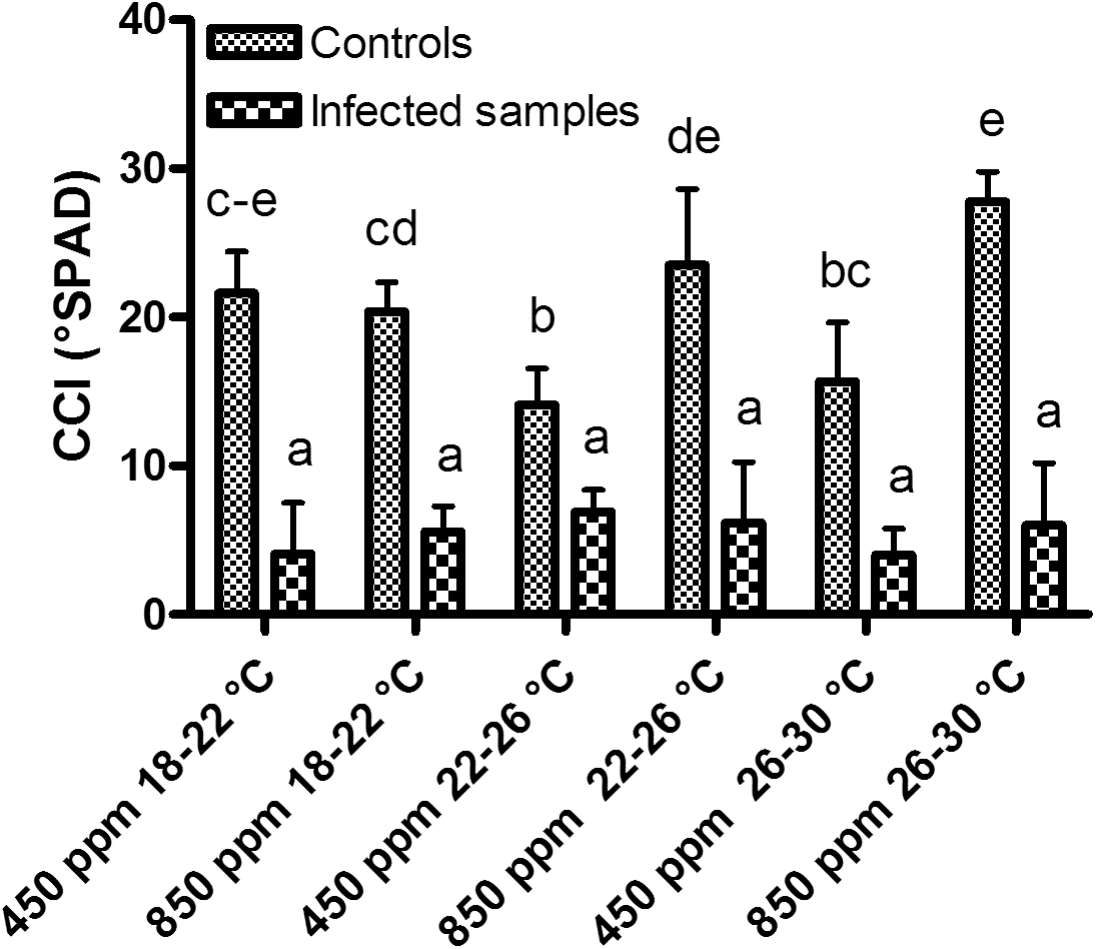
Assessment of chlorophyll content. Effect of different CO_2_ and temperature combinations on the chlorophyll content of the leaves (CCI, °SPAD) of rocket plants grown in a substrate artificially infested with *F*. *oxysporum* f.sp. *conglutinans* and the control. Tukey's HSD test (P < 0.05).

Similar trends were also observed for the CCI measurements in the infected samples (Fig 2) where no significant differences were observed between the phytotrons (P < 0.05). The non-infected plants showed higher levels of chlorophyll content than the infected rocket plants. Furthermore, significantly lower values were measured in the non-infected plants in phytotrons 3 and 5 (400–450 ppm CO_2_, 22–26°C and 400–450 ppm CO_2_, 26–30°C, respectively) (P < 0.05). Overall, the physiological measurements have confirmed that the disease development led to a remarkable decrease in the physiological performances of the leaves.

### Effect on root exudates

The results pertaining to the root exudates (Table 2) showed a similar trend for TOC and amino acids (AA), when the exudates from the infected roots and the control plants were compared. In both cases, when the temperature was lower (18–22°C), an increased concentration was registered in the samples from the infected plants. At 22–26°C and 26–30°C, the TOC and AA concentrations significantly increased for higher CO_2_ levels (P < 0.05). No significant differences were observed between the infected and non-infected plants for lower CO_2_ concentrations. The root exudates from the infected samples did not show any significant differences in TOC concentration at 400–450 ppm of CO_2_; conversely, an increasing trend of TOC concentration was registered when the temperature and CO_2_ level were increased (P < 0.05).

**Table 2:** Main root exudate components analyzed on rocket plants cultivated in infested and control substrates collected at the end of the cycle.

	Parameters	Phytotron^a^					
		1	2	3	4	5	6
		(400-450ppm CO_2_ 18–22°C)	(800-850ppm CO_2_ 18–22°C)	(400-450ppm CO_2_ 22–26°C)	(800-850ppm CO_2_ 22–26°C)	(400-450ppm CO_2_ 26–30°C)	(800-850ppm CO_2_ 26–30°C)
Infested samples	pH	7.23 ± 0.15 ab	7.38 ± 0.32 ab	7.10 ± 0.09 ab	7.65 ± 0.55 b	7.36 ± 0.19 ab	7.35± 0.23 ab
	Amino acids (mM g^-1^)	1.57 ±0.04 ab	1.92 ± 0.18 a-c	1.92 ± 0.28 a-c	2.78 ± 0.18 a-c	2.00 ± 0.17 a-c	4.97 ±0.66 e
	Organic acids (mM g^-1^)	1.90 ± 0.14 a	2.35 ± 0.12 a-c	5.86 ± 0.14 e	7.39 ± 0.42 f	1.54 ± 0.18 a	3.01 ± 0.33 cd
	TOC (mg g^-1^)	57.2 ± 1.09 a-c	50.6 ± 2.97 a	67.1 ± 3.17 de	77.8 ± 5.80 ef	57.2 ± 1.09 ab	98.7 ± 3.06 g
Controls	pH	6.83 ± 0.10 a	7.08 ± 0.13 ab	7.01 ± 0.12 ab	7.29 ± 0.07 ab	7.26 ± 0.11 ab	7.22 ± 0.14 ab
	Amino acids (mM g^-1^)	2.27 ±0.24 a-c	3.20 ± 3.14 a-d	2.19 ± 0.26 a-c	1.54 ± 0.03 a	2.08 ± 0.35 a-c	2.43 ±0.46 b-d
	Organic acids (mM g^-1^)	1.99 ± 0.07 ab	5.72 ± 0.27 e	8.07 ± 0.68 f	2.90 ± 0.22 b-d	3.57 ± 0.41 d	2.37 ± 0.38 a-c
	TOC (mg g^-1^)	74.0 ± 3.89 de	89.3 ± 5.58 fg	58.0 ± 2.69 a-c	46.0 ± 3.70 a	56.1 ± 3.82 a-c	65.1 ± 6.22 b-d

^a^ The root exudate parameters were expressed as the mean values of three plants for three cultivation cycles. Values with different superscripts in the same row differ significantly according to Tukey's HSD test (P<0.05)

The amino acid levels from the infected plants were not different at lower CO_2_ levels; conversely, higher CO_2_ levels only induced a significant increase in amino acids at high temperatures (P < 0.05).

The organic acids, in general, showed a different trend. When CO_2_ and temperature were kept low, no significant differences were registered between the treatments or in the phytotrons. However, increasing the concentration of CO_2_ led to an increased production of organic acids, but only in the control samples. Furthermore, at 22–26°C, for both levels of CO_2_, the organic acid concentration increased for all the samples. In general, the organic acid concentrations of all the samples were always significantly higher for higher CO_2_ levels (P < 0.05).

### Microbial analysis of substrate samples

The results of the bacterial and fungal plate counts on specific media are shown in Tables 3, 4 and 5. The plate counts of mesophilic bacteria (TBC) (Table 3) from infested substrate samples were not affected by the different CO_2_ and temperature levels. A significant decreasing trend was observed from time 0 up to 120 days for all the conditions considered (P < 0.05). Furthermore, the samples at 120 days were not significantly different between the phytotrons. The control substrate samples showed a similar trend to that observed in the infested samples. However, the samples taken after 120 days in phytotrons 2 and 4 (800–850 ppm CO_2_, 18–22°C and 800–850 ppm CO_2_, 22–26°C) were significantly higher (P < 0.05), that is, 7.17 and 6.87 Log CFU g^-1^, respectively.

**Table 3:** Total bacterial counts (TBC) of mesophilic bacteria from the infested and control substrate samples following incubation in phytotrons for 120 days.

	Sampling day	Phytotron (Log CFU g^-1^ ± SD^a^)					
		1	2	3	4	5	6
		(400-450ppm CO_2_ 18–22°C)	(800-850ppm CO_2_ 18–22°C)	(400-450ppm CO_2_ 22–26°C)	(800-850ppm CO_2_ 22–26°C)	(400-450ppm CO_2_ 26–30°C)	(800-850ppm CO_2_ 26–30°C)
Infested samples	0	7.38 ± 0.07 e	7.08 ± 0.11 c-e	7.19 ± 0.02 de	6.86 ± 0.03 b-e	7.05 ± 0.02 c-e	7.09 ± 0.02 c-e
	60	6.36 ± 0.10 ab	7.01 ± 0.05 c-e	6.80 ± 0.17 a-d	6.77 ± 0.07 a-d	6.56 ± 0.17 a-c	6.32 ± 0.28 ab
	120	6.45 ± 0.35 ab	6.43 ± 0.13 ab	6.54 ± 0.09 a-c	6.30 ± 0.52 a	6.34 ± 0.09 ab	6.26 ± 0.10 a
Controls	0	7.15 ± 0.08 c-e	7.39 ± 0.09 de	7.60 ± 0.02 e	7.19 ± 0.11 c-e	7.46 ± 0.02 de	7.22 ± 0.04 c-e
	60	6.40 ± 0.35 a	7.19 ± 0.18 c-e	6.62 ± 0.28 ab	7.13 ± 0.09 cd	6.91 ± 0.28 bc	7.57 ± 0.23 de
	120	6.26 ± 0.15 a	7.17 ± 0.07 c-e	6.42 ± 0.07 a	6.87 ± 0.10 c	6.57 ± 0.07 ab	6.17 ± 0.06 a

^**a**^The plate counts were calculated as the mean Log counts of the three replicates. Values with different superscripts differ significantly according to Tukey's HSD test (P<0.05)

**Table 4:** Total fungi community counts (TFC) from the infested and control substrate samples following incubation in phytotrons for 120 days.

	Sampling day	Phytotron (Log CFU g^-1^ ± SD^a^)					
		1	2	3	4	5	6
		(400-450ppm CO_2_ 18–22°C)	(800-850ppm CO_2_ 18–22°C)	(400-450ppm CO_2_ 22–26°C)	(800-850ppm CO_2_ 22–26°C)	(400-450ppm CO_2_ 26–30°C)	(800-850ppm CO_2_ 26–30°C)
Infested samples	0	6.59 ± 0.36 g-i	6.19 ± 0.23 f-h	7.13 ± 0.06 i	6.43 ± 0.11 g-i	5.92 ± 0.11 e-g	6.88 ± 0.08 hi
	60	5.12 ± 0.20 a-d	5.30 ± 0.24 b-e	5.47 ± 0.05 c-e	5.56 ± 0.14 d-f	5.06 ± 0.72 a-d	5.35 ± 0.15 b-e
	120	4.65 ± 0.07 ab	4.77 ± 0.10 a-c	4.81 ± 0.04 a-c	4.42 ± 0.16 a	4.84 ± 0.13 a-c	4.80 ± 0.06 a-c
Controls	0	6.52 ± 0.09 f	6.69 ± 0.40 f	6.30 ± 0.13 f	6.23 ± 0.24 ef	6.30 ± 0.00 f	6.55 ± 0.30 f
	60	5.12 ± 0.18 bc	5.75 ± 0.07 de	5.08 ± 0.16 ab	5.45 ± 0.04 cd	5.08 ± 0.07 bc	5.08 ± 0.11 bc
	120	4.53 ± 0.07 a	4.89 ± 0.16 c-e	4.75 ± 0.13 ab	4.93 ± 0.21 a-c	5.00 ± 0.08 a-c	4.74 ± 0.14 ab

^**a**^The plate counts were calculated as the mean Log counts of the three replicates. Values with different superscripts differ significantly according to Tukey's HSD test (P<0.05)

**Table 5:** Plate counts of *Fusarium oxysporum* f.sp. *conglutinans* from the infested substrate samples following incubation in phytotrons for 120 days.

	Sampling day	Phytotron (Log CFU g^-1^ ± SD^a^)					
		1	2	3	4	5	6
		(400-450ppm CO_2_ 18–22°C)	(800-850ppm CO_2_ 18–22°C)	(400-450ppm CO_2_ 22–26°C)	(800-850ppm CO_2_ 22–26°C)	(400-450ppm CO_2_ 26–30°C)	(800-850ppm CO_2_ 26–30°C)
Infested samples	0	3.77 ± 0.04 ab	3.80 ± 0.05 ab	4.24 ± 0.00 a-d	3.73 ± 0.12 a	3.78 ± 0.08 ab	4.00 ± 0.18 a-c
	60	4.00 ± 0.10 a-c	4.89 ± 0.02 e	4.46 ± 0.04 de	4.91 ± 0.15 e	4.06 ± 0.04 a-c	4.81 ± 0.10 de
	120	4.11 ± 0.04 a-c	4.38 ± 0.19 b-e	4.34 ± 0.74 a-e	4.32 ± 0.10 bc	4.18 ± 0.19 a-c	3.87 ± 0.11 a-c

^**a**^The plate counts were calculated as the mean Log counts of the three replicates. Values with different superscripts differ significantly according to Tukey's HSD test (P<0.05)

The total fungal community plate counts (TFC, Table 4) from the infested samples mirrored the bacterial counts; a significant decreasing trend (P < 0.05) was observed over time from time 0, without any significant differences between the phytotrons at 120 days. The control substrate sample counts were similar to the infested ones, with the exception of those from phytotron 2 (18–22°C and 850 ppm CO_2_) at 120 days, when the statistical values were higher (4.89 Log CFU g^-1^) than in the other conditions. However, (among fungal community) *Fusarium oxysporum* f.sp. *conglutinans* development was observed over time on the Komada medium supplemented with benomyl in the infested substrate samples. In general, most of the fungal community was represented by the pathogen, which developed over time and almost completely covered the total fungal community values up to the last survey (120 days). In particular, a general increasing trend was observed for all the conditions from time 0 in the phytotrons compared to time 120. Although no significant differences between phytotrons were observed at 120 days. A significant increasing trend from time 0 was observed for phytotrons 2 and 4 for high CO_2_ conditions in the last survey (from 3.80 to 4.38 and from 3.73 to 4.32 Log CFU g^-1^, respectively) (P < 0.05). With the exception of 26–30°C, high concentrations of CO_2_ were shown to positively affect the growth of *Fusarium oxysporum* f.sp. *conglutinans*. No pathogen was detected in the control substrate samples.

### DGGE analysis of the fungal microbiota

**Fig 3:**
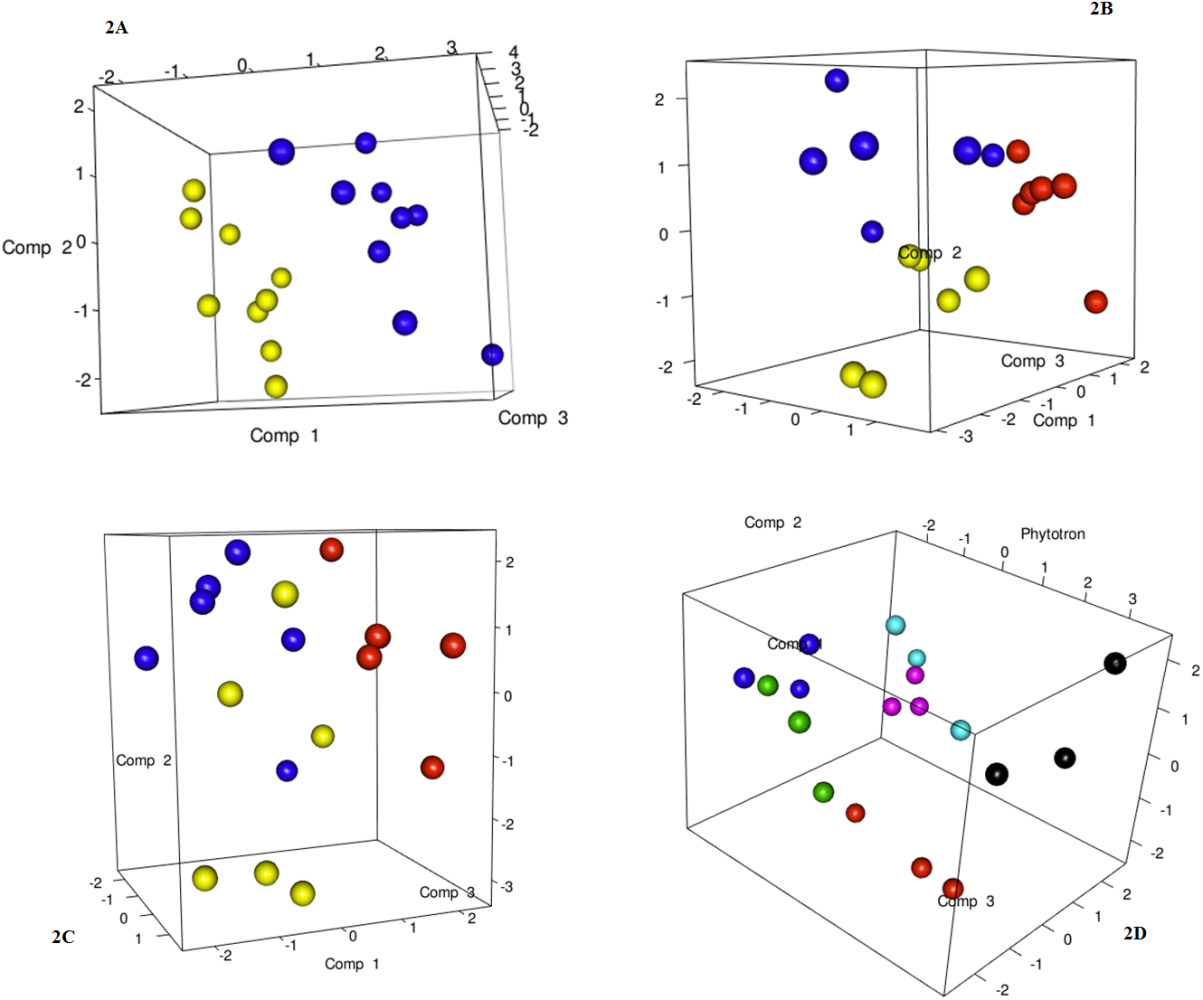
PLS-DA models based on DGGE similarity distance matrix. Plot A, PLS-DA models based on the DGGE similarity matrix as a function of CO_2_: 400–450 ppm (blue), 800–850 ppm (yellow); Plot B, PLS-DA models based on the DGGE similarity matrix as a function of the temperature: 26°C (blue), 22°C (yellow), and 30°C (red): Plot C, PLS-DA models based on the DGGE similarity matrix as a function of the time: time 0 (red), time 60 (yellow) and time120 (blue); Plot D; PLS-DA models based on the DGGE similarity matrix as a function of the phytotrons: 1 (red), 2 (yellow), 3 (blue), 4 (black), 5 (green), 6 (pink).

The PCR-DGGE fingerprints of the fungal community obtained from the DNA extracted directly from the infested samples in all the adopted conditions are presented in Fig 3. Repeated DNA extraction and PCR-DGGE analysis confirmed the results of the fingerprinting.

The ITS based DGGE analysis showed an average result of 11.7 bands per sample (min 3, max 17), (S1 Fig). The similarity matrix generated through the PyElph software was used to build a PLS-DA, as a function of the CO_2_ concentration, and the results showed a clear separation between samples at 850 and samples at 450 ppm CO_2_ (Fig 3A). Regardless of the temperature, the PLS models showed a clear separation of the samples (Fig 3B). When the samples were grouped on the basis of the sampling time, the samples at time 0 were clearly separated from the other samples (Fig 3C). The PLS-DA analysis, as a function of the phytotrons (Fig 3D), resulted in a certain degree of separation. The separation was particularly important for the samples from phytotron 4, which appear to group together and to be separated from the other phytotrons.

Furthermore, a binary band-matching table was analyzed in order to calculate the index of diversity (Shannon-Wiener diversity index *H’*). The results, using only the data from the ITS region, revealed, on the basis of their phytotron origin, that there was a biological diversity in the samples. In particular, the *H’* index and richness of the fungal community changed according to the environmental conditions that were set, and ranged from 1.61 to 2.83 and 5 to 17, respectively. At the end of the experiment, the *H’* index (1.79) and richness (10) were lower for phytotrons 4 and 6 (22–26 and 26–30°C at 850 ppm CO_2_), and the H’ index (2.83) and richness (17) were higher for Phytotron 1 (18–22°C and 450 ppm CO_2_). Higher CO_2_ and temperature were shown to be selective and to reduce fungal community diversity.

## Discussion

Plants that grow under elevated temperature and CO_2_ concentrations often exhibit positive responses by increasing photosynthesis and/or water and nutrient use efficiency [43]. However, how increases in CO_2_ might affect plant-pathogen interaction has not been addressed or debated to any great extent. The aim of this work was to evaluate the effect of simulated climate changes, by increasing CO_2_ concentrations and temperature levels, on the severity of *Fusarium oxysporum* f.sp. *conglutinans* on rocket plants. Phytotron chambers have already been used in several studies, since they provide total control of the environmental conditions and reproducible data in diverse pathosystems [20, 14–16]. Climate change is an on-going phenomenon, which affects whole ecosystems and causes multitrophic interactions that are difficult to understand [1]. The possibility of reproducing different climatic conditions by coupling carbon dioxide and temperature levels can/has been considered be very useful to obtain a better understanding of plant-pathogen interactions, as has been done in the present work which deals with a soilborne pathogen. Focusing on plant-soil systems, it is well known that plants directly or indirectly control and influence the multitrophic interactions of the soil through root exudates [22]. The results reported in this study refer to disease incidence, cultivable abundance, leaf physiological performances, root exudate analyses, and variability in substrate fungal profiles by PCR-DGGE. Several studies have reported that rising CO_2_ concentrations can cause plants to modify their root architecture and exudation compounds in the rhizosphere [44,45]. In addition, it has been predicted that global warming will directly influence multitrophic interactions if the soil temperature exceeds its buffering capacity and this could be followed by changes in the quality, quantity and diversity of plant and soil microbial communities and therefore plant pathogen development [1]. In the present study, a good disease level was reached under artificial inoculation of *F*. *oxysporum* f.sp. *conglutinans*, thus making it possible to evaluate the effect of the different climate change simulations. The temperatures and CO_2_ combinations in the six environmental conditions had a significant influence on the incidence of *Fusarium* wilt. Higher CO_2_ concentrations and temperatures caused a significant increase in DI, in particular in the 22–26°C range (Phytotron 4, Table 1). The effect of increased CO_2_ concentrations was still observed at 26–30°C on disease severity development, although lower values were observed than in the 22–26°C range, probably due to the suboptimal range of temperature that limits pathogen development. Although elevated CO_2_ had a significant effect on *Fusarium* wilt incidence, the increase in temperature also had a significant effect on disease development, particularly at 22–26°C, which is a favourable range of temperatures for disease development [30]. As previously reported, higher temperature or CO_2_ levels generally correspond to a greater severity of *Fusarium* wilt in other crops such as lettuce [16,46] or wheat [47]. As reviewed by Ainsworth and Rogers [8], rising CO_2_ concentrations and temperatures could influence leaf physiology, morphology and indirectly crop production by increasing or blocking their metabolic performances and photosynthetic efficiency. However, no relevant effects linked to disease development or environment conditions have been observed on PI and CCI data for infested and control plants, respectively. In fact, in infected rocket plants, large reductions of both indices have been recorded compared to controls, thus suggesting the high susceptibility of the photosynthetic machinery already at lower disease severity values. Furthermore, photosynthetic efficiency and leaf chlorophyll content are indicators of photosynthetic activity and chlorophyll stability. Fluorimeters and SPAD chlorophyll meters are frequently used for the measurement of foliar damage provoked by different biotic and abiotic stresses [35,48], although they were not considered useful for the present investigation.

Recent research in plant biology has pointed out the importance and the role of root exudates in mediating biological interactions in the rhizosphere. The chemical components of root exudates could deter or attract an organism with different effects on the plants. This would be particularly important during the pathogenesis of root-infecting fungal pathogens. However, the signalling and composition of root exudates in plant-pathogen interactions have not yet been elucidated [49–51]. To the best of the authors’ knowledge, this is the first report to have been made on the analysis of root exudates on wilted rocket plants under simulated climate change conditions. The results have shown a different exudation pattern between the controls and infected plants. Interestingly, higher levels of pH, organic acid and TOC (mainly composed of sugars) were observed in the infected samples in phytotron 4 where the disease index was higher. In addition, high values of organic acid, TOC and amino acids were also recorded in phytotron 6, where a consistent level of DI was observed. These results suggest the combined temperature and high CO_2_ effect on root component release. In particular, the TOC compounds underwent a significant increase, since they represent a carbon source for microbial metabolism and energy that in turn could influence the attraction and severity of the soilborne pathogen. Similarly, Kerks et al. [52] reported the activation of root exudate chemotaxis and pathogenicity genes of *S*. *enterica* serovar Typhimurium in lettuce, which are involved in root attachment and subsequent colonization. Although a great deal of information is available concerning the relationships between symbionts and plants, limited knowledge exists about the communication between plants and root pathogens mediated by rhizodeposits and/or by pathogen metabolite production (e.g. volatile organic compounds, VOCs) [53]. Further studies are needed to increase analytical skills and to analyse the chemistry of root exudates, and thus to resolve the dialogue that takes place between pathogens and plant roots. In addition, root exudates represent the main source of soil organic carbon, defined as soluble low-molecular weight components that are mainly composed of sugars, amino acids, organic acids and other secondary metabolites which vary from plant to plant and which are able to shape the rhizosphere microbiome [22]. Conversely, no relevant effects of CO_2_ or temperature levels have been observed in the control plants to explain the variation in the root exudate components in phytotron conditions, in line with what has been reported by Uselman et al. [54] in *Robinia pseudoacacia*.

It is well known that DGGE fingerprints can be used to describe microbial composition and diversity, but they do not provide any information on the abundance and concentration of separate microbial species [55]. For this reason, this limitation has partially been addressed here by conducting plate counts on several media. In the present experimental conditions, the total bacteria and fungi plate counts in both the infested and control substrate samples showed a decreasing trend over time up to the end of the experiment, without climate simulation effects being observed. These results suggested that the forecast increase in CO_2_ and temperature levels due to climate changes would have a limited effect on fungal and bacterial development. Recent reports [16,26,56] have shown similar results, while other studies have reported results that depend to a great extent upon the host, microorganisms and environment [57]. Conversely, the *F*. *oxysporum* f.sp. *conglutinans* plate counts at the end of the present experiment have shown a slight increase or no significant variation for time 0, except for phytotron 4 at higher CO_2_ concentrations, where the development and disease incidence were significantly higher. In phytotron 6, where disease incidence was high, no significant development of pathogen over time was detected. This behaviour supports the hypothesis of a plant-mediated effect increasing disease incidence. The variability in the fungal profiles was assessed by means of the PCR-DGGE approach, which has frequently been used, with good results, in other simulated global change studies [16,26]. The samples were clearly separated in relation to the temperature or CO_2_. In addition, this result was further confirmed from an examination of the Shannon-Wiener indices. Interestingly, phytotrons 4 and 6 showed the least diversity and the highest DI. Thus, a low diversity might have a higher impact on disease development efficiency than the species visualized from the DGGE examination. The loss in diversity caused by higher temperature and CO_2_ levels could be due to a plant-mediated effect that causes the selection of a few dominant species and which may exclude others species through a competition strategy.

## Conclusions

The disease incidence of pathogenic *Fusarium* species could increase due to the effects of the global changes that have been predicted for the future. The present experimental conditions have shown a coupled temperature and CO_2_ effect on disease severity, which is probably plant-mediated, as reported for other pathosystems. Although no specific predictions can be made on field conditions, the data obtained from phytotron growth chambers could help to unravel the complexity of plant-soilborne pathogen interactions that take place under climate change conditions, in order to implement model prediction and prevention strategies.

## Supporting Information

S1 Fig Similarity dendrogram generated from the digitized PCR-DGGE fingerprints of DNA directly extracted from the substrate infested with *F*. *oxysporum* f.sp. *conglutinans*. (TIF)
