# Supplementary figures and images for: Effect of Elevated Atmospheric CO2 and Temperature on the Disease Severity of Rocket Plants Caused by Fusarium Wilt under Phytotron Conditions
Source: PLoS One. 2015 Oct 15;10(10):e0140769. doi: 10.1371/journal.pone.0140769 (PMC4607163; doi:10.1371/journal.pone.0140769)

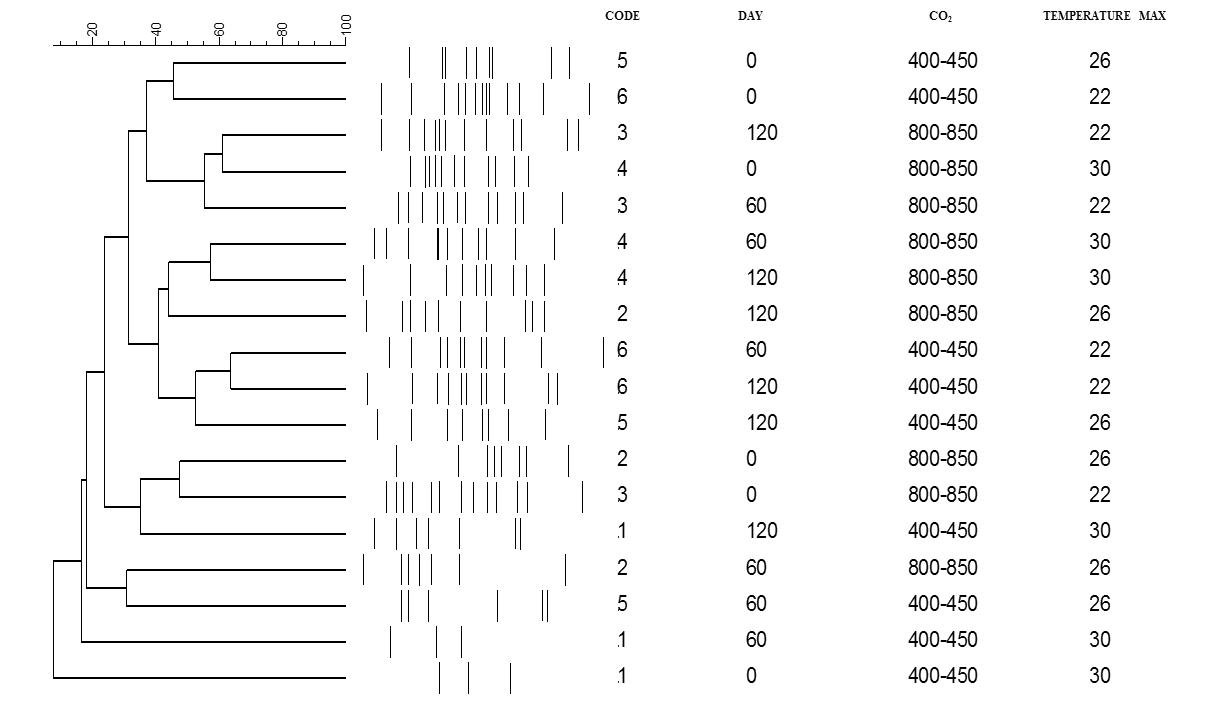

Supplement: S1 Fig — (TIF) [file pone.0140769.s001.tif]
